# Diisopropylamine Dichloroacetate, a Novel Pyruvate Dehydrogenase Kinase 4 Inhibitor, as a Potential Therapeutic Agent for Metabolic Disorders and Multiorgan Failure in Severe Influenza

**DOI:** 10.1371/journal.pone.0098032

**Published:** 2014-05-27

**Authors:** Kazuhiko Yamane, Irene L. Indalao, Junji Chida, Yoshikazu Yamamoto, Masaaki Hanawa, Hiroshi Kido

**Affiliations:** 1 Division of Enzyme Chemistry, Institute for Enzyme Research, The University of Tokushima, Tokushima, Japan; 2 R&D Department, Daiichi Sankyo Healthcare Co., Ltd., Tokyo, Japan; University of North Carolina at Greensboro, United States of America

## Abstract

Severe influenza is characterized by cytokine storm and multiorgan failure with metabolic energy disorders and vascular hyperpermeability. In the regulation of energy homeostasis, the pyruvate dehydrogenase (PDH) complex plays an important role by catalyzing oxidative decarboxylation of pyruvate, linking glycolysis to the tricarboxylic acid cycle and fatty acid synthesis, and thus its activity is linked to energy homeostasis. The present study tested the effects of diisopropylamine dichloroacetate (DADA), a new PDH kinase 4 (PDK4) inhibitor, in mice with severe influenza. Infection of mice with influenza A PR/8/34(H1N1) virus resulted in marked down-regulation of PDH activity and ATP level, with selective up-regulation of PDK4 in the skeletal muscles, heart, liver and lungs. Oral administration of DADA at 12-h intervals for 14 days starting immediately after infection significantly restored PDH activity and ATP level in various organs, and ameliorated disorders of glucose and lipid metabolism in the blood, together with marked improvement of survival and suppression of cytokine storm, trypsin up-regulation and viral replication. These results indicate that through PDK4 inhibition, DADA effectively suppresses the host metabolic disorder-cytokine cycle, which is closely linked to the influenza virus-cytokine-trypsin cycle, resulting in prevention of multiorgan failure in severe influenza.

## Introduction

Influenza A virus (IAV) is the most common infectious pathogen in humans and causes significant morbidity and mortality, particularly in infants and elderly population [1], [2]. Multiorgan failure (MOF) with severe edema is reported in the progressive stage of seasonal influenza virus pneumonia and influenza-associated encephalopathy, particularly in patients with underlying risk factors [3]–[5] and is common in the highly pathogenic avian IAV infection [6]. The antiviral neuraminidase inhibitors are used for treatment in the initial stage of IAV infection and a 5-day course of these compounds is recommended for individuals with flu symptoms of not more than 2 days. A proportion of individuals with progressive symptoms after the initial stage of infection develop MOF with metabolic disorders and vascular hyperpermeability. To date, the pathogenesis and treatment target molecules of MOF by IAV remain poorly understood.

We reported previously that the influenza virus–cytokine–trypsin cycle is one of the main underlying mechanisms of vascular hyperpermeability and MOF in severe influenza [7]. Severe influenza causes marked increases in the levels of proinflammatory cytokines, such as tumor necrosis factor (TNF)-α, interleukin (IL)-6, and IL-1β, coined the cytokine storm. These cytokines alter the cellular redox state through their receptors and reduce the expression of four complex I subunits, oxygen consumption [8], [9] and ATP synthesis in mitochondria, as well as increase mitochondrial O_2_- production and intracellular calcium concentration [Ca^2+^]_i_ [10]. ATP depletion dissociates zonula occludens-1, intracellular tight junction component, from the actin cytoskeleton and increases junctional permeability [11]. These cytokines also upregulate trypsin, which mediates the post-translational proteolytic cleavage of viral envelope hemagglutinin and is crucial for viral entry and replication cycle [12], in various organs and endothelial cells. Trypsin also increases [Ca^2+^]_i_ and enhances loss of zonula occludens-1 in cells via the protease-activated receptor (PAR)-2 [7], [13], [14]. In addition, we reported recently that metabolic disorders and energy crisis in brain endothelial cells by IAV infection are the main mechanisms of influenza-associated encephalopathy, which is characterized in infancy and early childhood in East Asian countries by persistently high fever and severe brain edema [5], [15], [16]. Patients with influenza-associated encephalopathy exhibit thermal instability of compound variants for [1055T>G/F352C] and [1102G>A/V368I] of carnitine palmitoyltransferase II (CPT II), resulting in secondary CPT II deficiency and mitochondrial energy crisis through disorders of long-chain fatty acid metabolism during hyperpyrexia. These findings indicate that MOF is the final outcome of metabolic and mitochondrial fuel disorders in severe influenza, although the precise signaling pathways involved in these disorders are still unknown.

The mitochondrial pyruvate dehydrogenase (PDH) complex (PDC) catalyzes the oxidative decarboxylation of pyruvate, linking glycolysis to the energetic and anabolic functions of the tricarboxylic acid cycle and fatty acid synthesis via acetyl-CoA. Therefore, PDC is a key enzyme for regulation of whole body glucose, lipid, lactate and ATP homeostasis. PDC is a structurally complex enzyme of three components [PDH (E1), dihydrolipoamide acetyltransferase (E2), and dihydrolipoamide dehydrogenase (E3)] with two specific regulatory enzymes, pyruvate dehydrogenase kinases (PDKs) 1–4 for phosphorylation (inactivation) of the α-subunit of PDH and pyruvate dehydrogenase phosphate phosphatases (PDPs) 1 and 2, for dephosphorylation (activation, reactivation) of the α-subunit of PDH [17], [18]. Expression of PDKs is linked to homeostasis of glucose, lipid, lactate, ATP, cytokines, and hormone levels in various diseases and suppressively regulates PDH activity [17]–[20].

In the present study, we describe selective and marked up-regulation of PDK4, an isoform of PDK, together with down-regulation of PDH activity and ATP levels in the skeletal muscles, heart, liver and lungs, but not the brain, in mice with the progressive stage of severe influenza and cytokine storm conditions. The results also showed that diisopropylamine dichloroacetate (DADA), which is the active component of pangamic acid [21] and commercially available as a Liverall (Daiichi Sankyo Co., Tokyo, Japan) for over 50 years for the treatment of chronic liver diseases, is a safe inhibitor of PDK4. DADA selectively and effectively inhibited PDK4, resulting in significant restoration of PDH activity as well as various metabolic disorders, such as ATP levels in various organs, and improved glucose, lactate and β-hydroxybutyric acid levels in the blood. Amelioration of PDH suppression in the infected mice by DADA was associated with significant vital improvements, such as restoration of energy metabolism, suppression of cytokine storm and viral proliferation, with marked improvement of survival.

## Methods

### Reagents

DADA was obtained from Daiichi Sankyo Healthcare Co. (Tokyo). Sodium DCA was purchased from Wako Pure Chemical Industries (Osaka, Japan).

### Animals and virus

All animals were treated according to the Guide for the Care and Use of Laboratory Animals (NIH Publication No. 85–23, 1996), and the study was approved by the Animals Care Committee of the University of Tokushima. Specified pathogen-free 4-week-old weanling C57BL/6CrSlc (B6) female mice were obtained from Japan SLC and maintained at 12-h light/dark cycle in a temperature-controlled room with free access to food and water. IAV/PR/8/34(H1N1) was kindly provided by The Research Foundation for Microbial Diseases of Osaka University (Kagawa, Japan). Under ketamine and xylazine anesthesia, 60, 120 and 200 plaque-forming units (pfu) of IAV/PR/8/34(H1N1) in 15 µL of saline or saline alone as non-infected control was instilled intranasally in mice. Mice were treated orally with DADA, a PDK inhibitor, starting immediately after infection at 50 mg/kg at 12-h intervals for 14 days in 100 µL sterilized 0.5% methylcellulose 400 (MC) solution mixed with calcium gluconate, a food additive, in commercially available medicine format at 4.68 mg/mL. The dose of DADA was adjusted to yield daily molar doses similar to the range of DCA used clinically (clinical doses, 25–100 mg/kg/day [22], [23]). Positive control mice were treated with intraperitoneal injection of 100 µL of DCA at 28 mg/kg, an equivalent molar dose to DADA, at 12-h intervals daily. Mice were monitored daily for body weight and food and water intake and assessed visually for signs of clinical diseases including inactivity, ruffled fur, labored respiration and huddling behavior. Mice that lost ≥30% of their original body weight and/or displayed evidence of pneumonia were euthanized by overdose of intraperitoneal injection of ketamine and xylazine.

### Blood glucose, lactate, free fatty acids and β-hydroxybutyric acid assays

Whole blood levels of glucose, lactate, and β-hydroxybutyric acid were measured according to the protocols recommended by Medisafe-Mini GR-102 (Terumo, Japan), Lactate-Pro LT-1710 (Arkray, Japan) and Precision Xceed (Abbot, Japan) hand-held meter, respectively. Serum levels of free fatty acids were measured according to the protocols provided with the Free Fatty Acid Quantification Kit (Abcam, Japan).

### Blood and tissues ATP assays

Tissue ATP levels were measured by the firefly bioluminescence assay kit with an improved phenol-based ATP extraction reagent [24] (AMERIC-ATP (T) kit; Wako Pure Chemical Industries) according to the protocol supplied by the manufacturer. Freshly prepared brain, heart, lungs, liver and skeletal muscle (gastrocnemius muscle) tissues were homogenized immediately with 3.0 mL of ice-cold phenol-based ATP extraction reagent by Ultra-Turrax (Ika Japan, Nara, Japan). The tissue ATP levels were normalized against wet-tissue weight. Blood ATP levels were also measured by AMERIC-ATP kit for blood and cells using the procedure described previously [25].

### *IC*_50_ analysis

The half maximal inhibitory concentrations (*IC*_50_) of DADA and DCA against human PDK2 and PDK4 recombinant proteins expressed baculovirus system were measured by the method of off-chip mobility shift assay using a panel of human recombinant active kinases [26] from Carna Biosciences, Inc. (Kobe, Japan).

### PDH activity

PDH activity was measured in the brain, heart, lungs, skeletal muscles, and liver by the PDH Enzyme Activity Microplate Assay Kit (MSP18) (MitoSciences, Eugene, OR). The PDH was immunocaptured within the microplate and activity was determined by following the reduction of NAD^+^ to NADH, coupled to the reduction in a reporter dye color. Tissues homogenates in PBS(-) were prepared by Dounce Tissue Grinder to avoid mitochondrial damage. Tissue samples were then solubilized and diluted with detergent in the assay kit at the optimal protein concentrations for each organ to immunocapture PDH on the microplate; brain (1 mg/well), heart (200 µg/well), lungs (1 mg/well), liver (800 µg/well) and muscles (1 mg/well). After adding the reaction mixture, absorbance was monitored at 450 nm for 15 min. The activity was expressed as the initial rate of reaction.

### Western immunoblotting

Anti-mouse PDK4 antibody (provided generously by Dr. M. Horiuchi, Kagoshima University, Japan), was used for detection of PDK4 in the heart, lungs, skeletal muscles and liver tissues [27]. Freshly isolated tissues were homogenized with 7 volumes of RIPA lysis buffer containing 50 mM Tris-HCl, pH 8.0, and 150 mM NaCl, 10% glycerol, 1% NP 40, 0.5% deoxycholate, 0.4 mM EDTA, and 0.5 mM sodium orthovanadate (Thermo Scientific, Yokohama, Japan) and centrifuged at 12,000×*g* for 20 min. The extracts (with protein concentration of 16.7 µg) were used for immunoblotting, as described previously [13]. Immunoreactive bands were visualized by enhanced chemiluminescence detection system (GE Healthcare, Tokyo) and the detected bands were quantified by ImageJ software.

### Real-time PCR

Total RNA was extracted from mice tissues with RNeasy Mini kit (Qiagen, Hilden, Germany) and reverse transcribed using oligo primers and universal primers in SuperScript III RT kit (Gibco BRL, Grand Island, NY) for cDNA synthesis. RT-PCR and quantification of gene expression by real-time PCR were performed using a Fast Start Universal SYBR Green Master (Roche Diagnostics, Mannheim, Germany) on an ABI Prism 7300 system. The primer pairs used to amplify influenza A virus NS1, PDK1–4, PDP 1–2, trypsin and mouse glyceraldehyde-3-phosphate dehydrogenase (GAPDH) are listed in Table 1. PCR was initiated at 95°C for 10 min to activate HotStartTaq DNA polymerase, followed by 40 cycles of 30-s denaturation at 95°C, 30-s annealing at 52°C and 50-s extension at 72°C.

**Table 1:** Sequences of primers used in real-time PCR.

Gene	Forward primer (5′-3′)	Reverse primer (5′-3′)
PDP1	CGGGCACTGCTACCTATAATT	ACAATTTGGACGCCTCCTTACT
PDP2	GGCTGAGCATTGAAGAAGCATT	GCCTGGATTTCTAGCGAGATGT
PDK1	CCGGGCCAGGTGGACTTC	GCAATCTTGTCGCAGAAACATAAA
PDK2	GCTTCCCCTGACCTGGAGAT	AGGCTGGACTCGGCTTT
PDK3	CGGTCCCCAAGCAGATCGA	GTTAGCCAGTCGCACAGGAG
PDK4	CACATGCTCTTCGAACTCTTCAAG	TGATTGTAAGGTCTTCTTTCCCAAG
Trypsin	AGTGGGTGGTGTCTGCAGCTCA	GATTCCTGCCAGGGTGACTC
NS-1	TACCTGCGTCGCGTTACCTAA	TGCTTCTCCAAGCGAATCTCT
GAPDH	CATCACCATCTTCCAGGA	GAGGGGGCCATCCACAGTCTTC

### Enzyme-linked immunosorbent assay (ELISA)

The lung samples were crushed with 3 mL PBS and IL-6, IL-2, TNF-α, IL-1β, and IFN-γ levels in the homogenates were measured using Quantikine ELISA kit (R&D systems, Minneapolis, MN). IFN-α and IFN-β levels were measured using VeriKine ELISA kits (R&D systems) according to the respective protocols provided by the manufacturer.

### Histological evaluation

After euthanasia, the lungs were isolated then fixed with 4% paraformaldehyde, dehydrated, infiltrated, and cut into 5-µm paraffin-embedded tissue sections for histological evaluation, as described previously [13]. Tissue sections were stained with hematoxylin–eosin.

### Statistical analysis

Results are presented as mean ± SD. Differences between groups were analyzed by one-way analysis of variance (ANOVA) with Tukey post hoc test. Survival rate was analyzed by the Kaplan-Meier and log-rank tests. Changes in body weight among groups were analyzed by two-way ANOVA. All statistical tests were performed using the Microsoft Excel software (Microsoft Corp, Redmond, WA) add-in Ekuseru-Toukei 2010 version 1.10 (Social Survey Research Information Co.). All *P* values are two-sided, and those less than 0.05 were considered statistically significant.

## Results

### Low PDH activity and ATP levels in various organs of mice infected with sub-lethal doses of IAV infection

Intranasal administration of 60 pfu of IAV PR/8/34(H1N1) in 15 µL of saline in 4-week-old B6 mice was semi-lethal by day 14 post-infection; the animals began to die after day 7 post-infection. Furthermore, 120 pfu was sub-lethal with 3–5% survival rate. Animals administered 200 pfu began to die after day 7 post-infection and all were dead (100% mortality rate) by day 10 (lethal dose). At day 4 post-infection and thereafter, the majority of animals infected with these doses of IAV developed clinical signs of infection (e.g., inactivity, loss of body weight, ruffled fur, labored respiration and huddling behavior). MOF starts at day 4 post-IAV-infection, and is characterized by marked increase in the levels of proinflammatory cytokines and a rapid increase in viral proliferation via the influenza virus–cytokine–trypsin cycle [7], [12]. After peak viral proliferation in the lung at days 4 to 5 post-infection, various metabolic disorders associated with cellular dysfunction become obvious in various organs, together with the initiation of host protective immunity [12].

**Figure 1:**
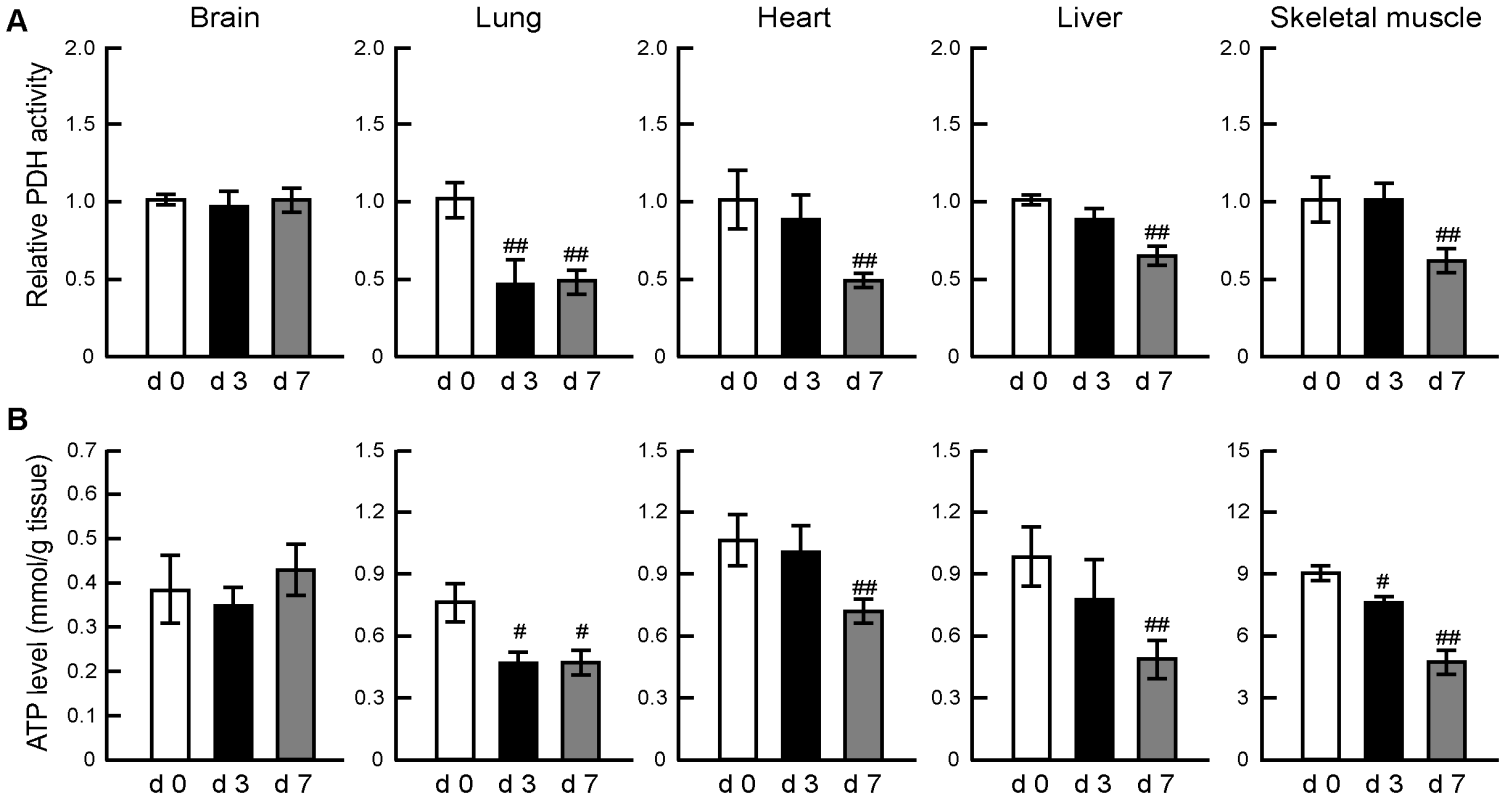
Time course of changes in PDH activity and ATP levels in the skeletal muscles, heart, lungs, liver and brain of IAV-infected mice. Mice were infected with IAV/PR/8/34(H1N1) at 120 pfu intranasally and the levels of PDH activity (A) and ATP (B) in the skeletal muscles, heart, lungs, liver and brain were analyzed at days 0 (d0), 3 (d3) and 7 (d7) post-infection. PDH activity levels after IAV infection relative to the values at day 0. Data are mean ± SD of 5 mice per group. ^#^ *P*<0.05, ^##^ *P*<0.01 vs. day 0, by one-way analysis of variance (ANOVA) with Tukey post hoc test.

Mice infected with 120 pfu of IAV had significantly low PDH activity in the lungs at day 3 post-infection (to about 50–60% of the non-infected control) and also in the skeletal muscles, liver and heart at day 7 post-infection (Figure 1A). On the other hand, PDH activity in the brain remained unchanged at days 3 and 7 post-infection. ATP levels in the heart, lungs, skeletal muscles and liver, but not in the brain, showed similar reduction patterns (both time course and tendency, Figure 1B). These findings indicate that sub-lethal doses of IAV induce significant disorder of glucose oxidation, reduction of energy metabolism, and poor ATP generation in the skeletal muscles, liver, lungs and heart, through reduction of PDH activity in the mitochondria.

### IAV infection induces marked and selective up-regulation of PDK4 in the skeletal muscles, heart, lungs and liver

**Figure 2:**
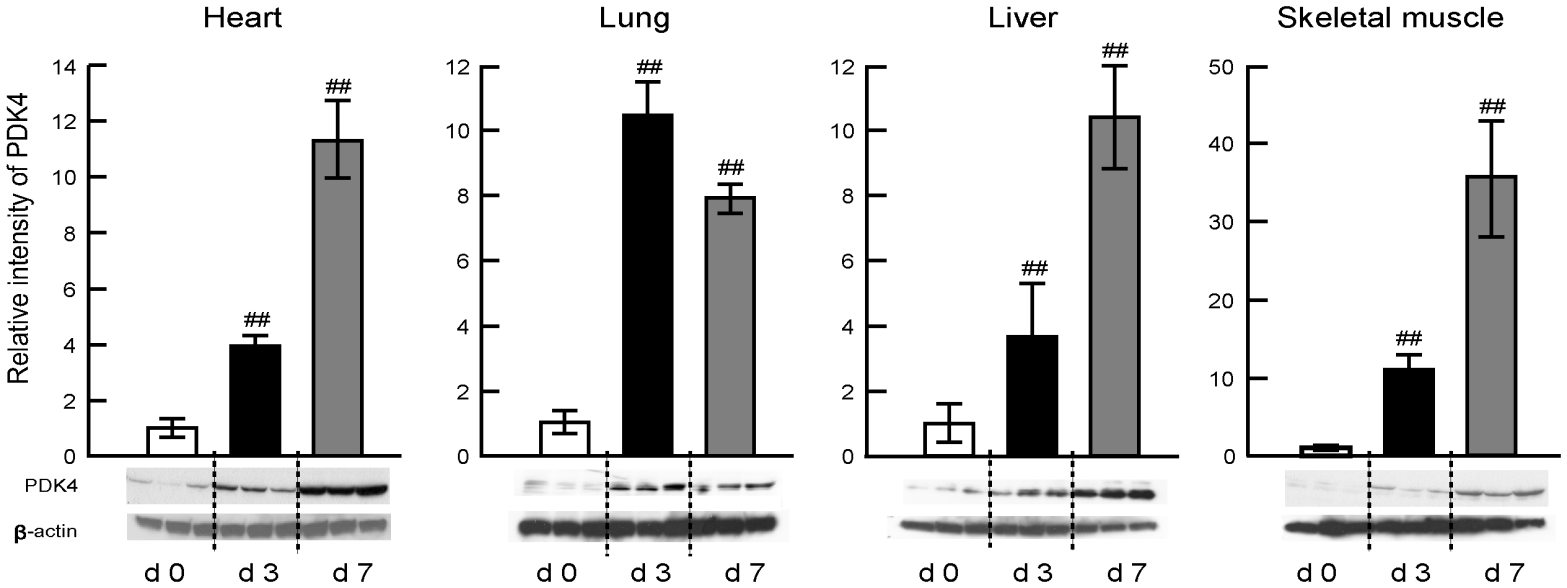
Time course of changes in PDK4 protein expression levels in the skeletal muscles, heart, lungs and liver of IAV-infected mice. Mice were infected with 120 pfu of IAV, and PDK4 protein expression levels in the skeletal muscles, heart, lungs and liver were measured by western immunoblotting at day 0 (d0), day 3 (d3) and day 7 (d7) post-infection. β-Actin was used as internal control. PDK4 expression levels after IAV infection relative to the values at day 0. Data are mean ± SD of three experiments from 5 mice per band. *P<0.05, **P<0.01, vs. day 0, by one-way analysis of variance (ANOVA) with Tukey post hoc test.

Oxidative decarboxylation of pyruvate involves three structurally complex enzymes, including PDH (E1), E2, and E3, that catalyze the conversion of pyruvate to acetyl-CoA [17], [18]. The complex also contains two specific phosphorylation-dephosphorylation enzymes; PDK and PDP. Table 2 shows changes in relative mRNA expression levels of these regulatory compounds in the liver, heart, lungs, skeletal muscles and brain at days 0, 3 and 7 post-infection. Although a sub-lethal dose of IAV infection was associated with variable changes in the expression of these phosphorylation-dephosphorylation enzymes, marked and predominant up-regulation of PDK4 was evident in the skeletal muscles, liver, lungs and heart. However, up-regulation of PDK4 in the brain was very mild even at day 7 post-infection. Western immunoblotting analysis (Figure 2) confirmed predominant up-regulation of PDK4 protein with a peak at day 3 post-infection in the lungs, and peaks at day 7 post-infection in the skeletal muscles, heart and liver. Changes in protein levels in these organs were almost identical to those in mRNA levels.

**Table 2:** Relative mRNA expression levels of PDPs and PDKs after severe IAV infection.

	Days after infection	Brain	Heart	Lung	Liver	Skeletal muscle
PDP1	Day 0	1.0±0.31	1.0±0.18	1.0±0.23	1.0±0.12	1.0±0.06
	Day 3	1.20±0.43	0.88±0.42	1.21±0.13	2.30±0.42##	0.72±0.21
	Day 7	0.68±0.24	0.66±0.20	0.43±0.11	2.77±0.22##	0.30±0.09##
PDP2	Day 0	1.0±0.37	1.0±0.12	1.0±0.12	1.0±0.18	1.0±0.18
	Day 3	2.0±0.52#	0.26±0.05##	1.51±0.56	2.19±0.24#	1.12±0.10
	Day 7	2.2±0.31#	0.20±0.06##	0.76±0.09	1.78±0.31#	2.17±0.27##
PDK1	Day 0	1.0±0.06	1.0±0.27	1.0±0.21	1.0±0.13	1.0±0.09
	Day 3	1.25±0.40	0.87±0.21	0.69±0.17	0.85±0.49	0.82±0.21
	Day 7	1.38±0.34	0.81±0.18	0.69±0.21	0.98±0.43	0.60±0.12#
PDK2	Day 0	1.0±0.06	1.0±0.18	1.0±0.23	1.0±0.12	1.0±0.18
	Day 3	1.56±0.34	0.93±0.43	0.40±0.11	2.05±0.37#	1.12±0.12
	Day 7	1.76±0.21	1.25±0.61	0.15±0.04	1.25±0.14	1.18±0.20
PDK3	Day 0	1.0±0.37	1.0±0.06	1.0±0.08	1.0±0.18	1.0±0.12
	Day 3	1.23±0.34	1.02±0.24	1.16±0.43	3.55±0.49##	0.68±0.18
	Day 7	1.01±0.24	1.07±0.15	1.01±0.09	4.68±0.31##	0.75±0.20
PDK4	Day 0	1.0±0.06	1.0±0.07	1.0±0.15	1.0±0.37	1.0±0.34
	Day 3	1.26±0.21	6.57±1.34##	5.01±1.23##	1.94±0.11	12.46±5.46##
	Day 7	1.37±0.1#	11.59±0.24##	3.59±1.34##	7.97±1.60##	85.32±7.99##

Data are mean ± SD.

# *P*<0.05,

## *P*<0.01, vs. before infection (Day 0) for each organ, by one-way analysis of variance and Turkey post hoc test.

### DADA as a novel PDK4 inhibitor

A sub-lethal dose of IAV up-regulates PDKs, particularly PDK4, resulting in the suppression of PDH activity in the mitochondria (Table 2, Figures 1 and 2). Among the known synthetic inhibitors of PDK [28], such as dichloroacetate (DCA), AZD7545 and radicicol, the pyruvate analog DCA is the most common classic inhibitor for PDK isoforms [29]. In the present study, we analyzed the inhibitory activity of DADA, a DCA derivative and commercially-available safe compound, against PDK4 and PDK2, which are the main PDK isoforms in the skeletal muscles, heart, lungs and liver [20], and used DCA as a positive control in these experiments (Table 3). Kinetic studies showed that the *IC*_50_ values, i.e., DADA concentrations at which the reaction rates are suppressed by 50%, were 50.9 µM against PDK4 and 636.0 µM against PDK2. These values were almost identical to those of DCA against PDK4 and PDK2, respectively. These results indicate that DADA is a novel PDK4 inhibitor with about 12.5-fold higher affinity than that against PDK2.

**Table 3:** DCA- and DADA-induced percent inhibition and related *IC_50_* values (µM).

		DCA			DADA		
		100 µM	300 µM	1000 µM	100 µM	300 µM	1000 µM
% inhibition	PDK2	9.0±0.3	33.0±0.18	58.4±3.3	16.6±0.2	37.8±0.2	57.2±4.1
	PDK4	63.9±3.3	88.0±1.0	97.8±12	70.2±2.1	93.4±10	99.1±13
*IC_50_* (µM)	PDK2		676.0			636.0	
	PDK4		57.8			50.9	

Data are mean ± SD.

### DADA significantly increases PDH activity and ATP levels in the skeletal muscles, heart, lungs and liver

**Figure 3:**
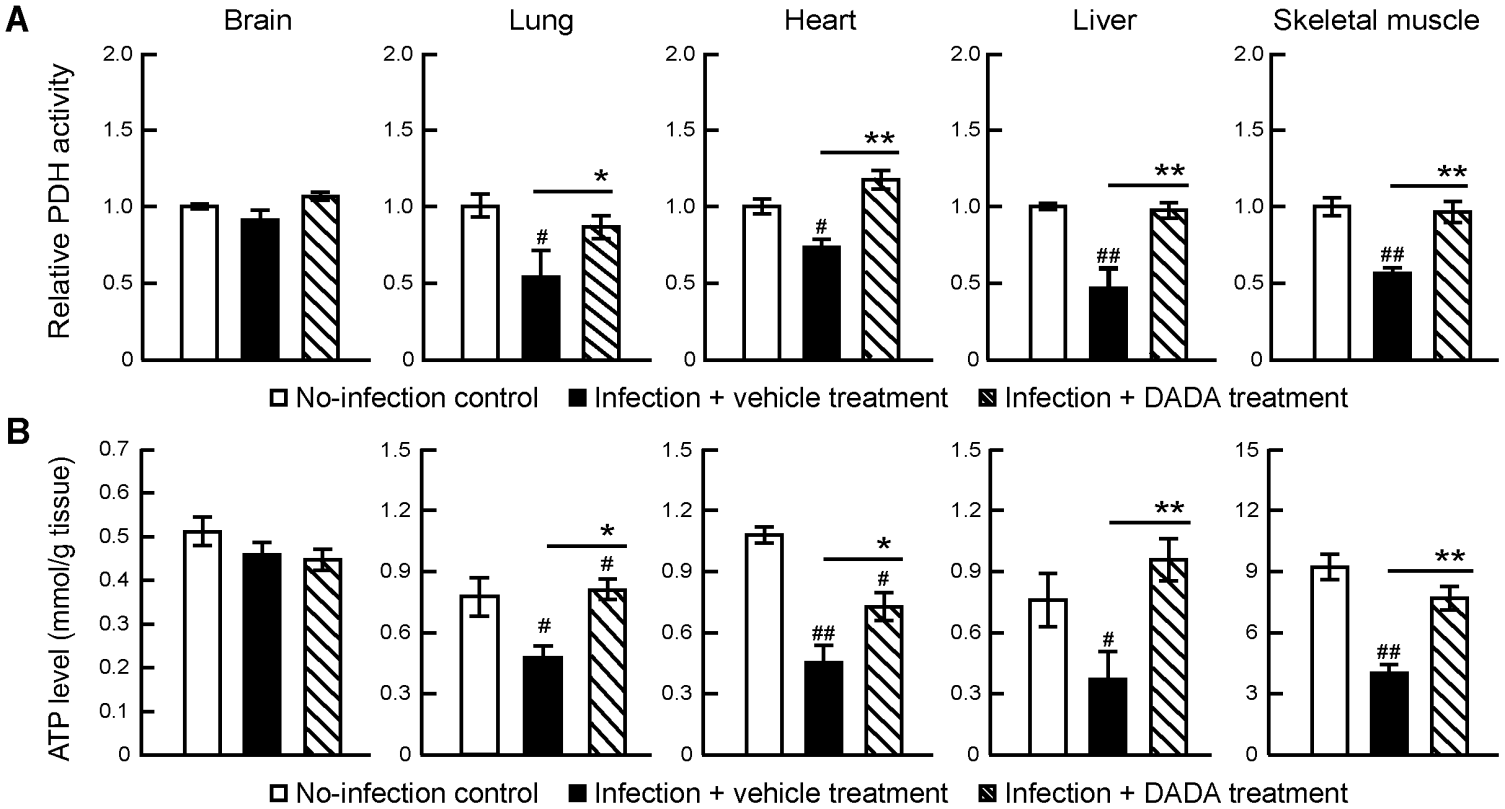
Treatment with DADA restores suppressed PDH activity and ATP levels in the skeletal muscles, heart, lungs and liver of IAV-infected mice. Mice infected with 120 pfu of IAV were treated orally with DADA at 50 mg/kg or vehicle at 12-h intervals for 14 days, and the levels of PDH activity (A) and ATP (B) in the skeletal muscles, heart, lungs, liver and brain of mice were analyzed at day 7 post-infection. PDH activity levels relative to the values of the control (no-infection). Values are mean ± SD of 5 mice per group. ^#^ *P*<0.05, ^##^ *P*<0.01, vs. no-infection, *P<0.05, **P<0.01, vs. infected group treated with vehicle, by one-way analysis of variance (ANOVA) and Tukey post hoc test.

To analyze the effects of DADA as a PDK4 inhibitor on PDH activity and ATP levels in different tissues, mice infected with a sub-lethal dose of IAV PR/8/34(H1N1) were treated orally with DADA immediately after the infection, at 50 mg/kg twice daily. As described above, the infection resulted in marked suppression of PDH activities and ATP levels at day 7 post-infection, the time point before animal death, in the skeletal muscles, liver, lungs and heart to about 25–52% and 48–63% of the non-infected control, respectively (Figure 3). DADA significantly prevented the effects of IAV infection and restored PDH activities to levels similar to those recorded before infection. DADA also increased ATP levels to 68–130% of non-infected control in the skeletal muscles, heart, lungs and liver. On the other hand, PDH activity and ATP levels in the brain were neither affected by the infection nor DADA treatment.

### DADA tends to normalize biased blood glucose, lactate, β-hydroxybutyric acid, free fatty acids, and ATP levels after infection

**Figure 4:**
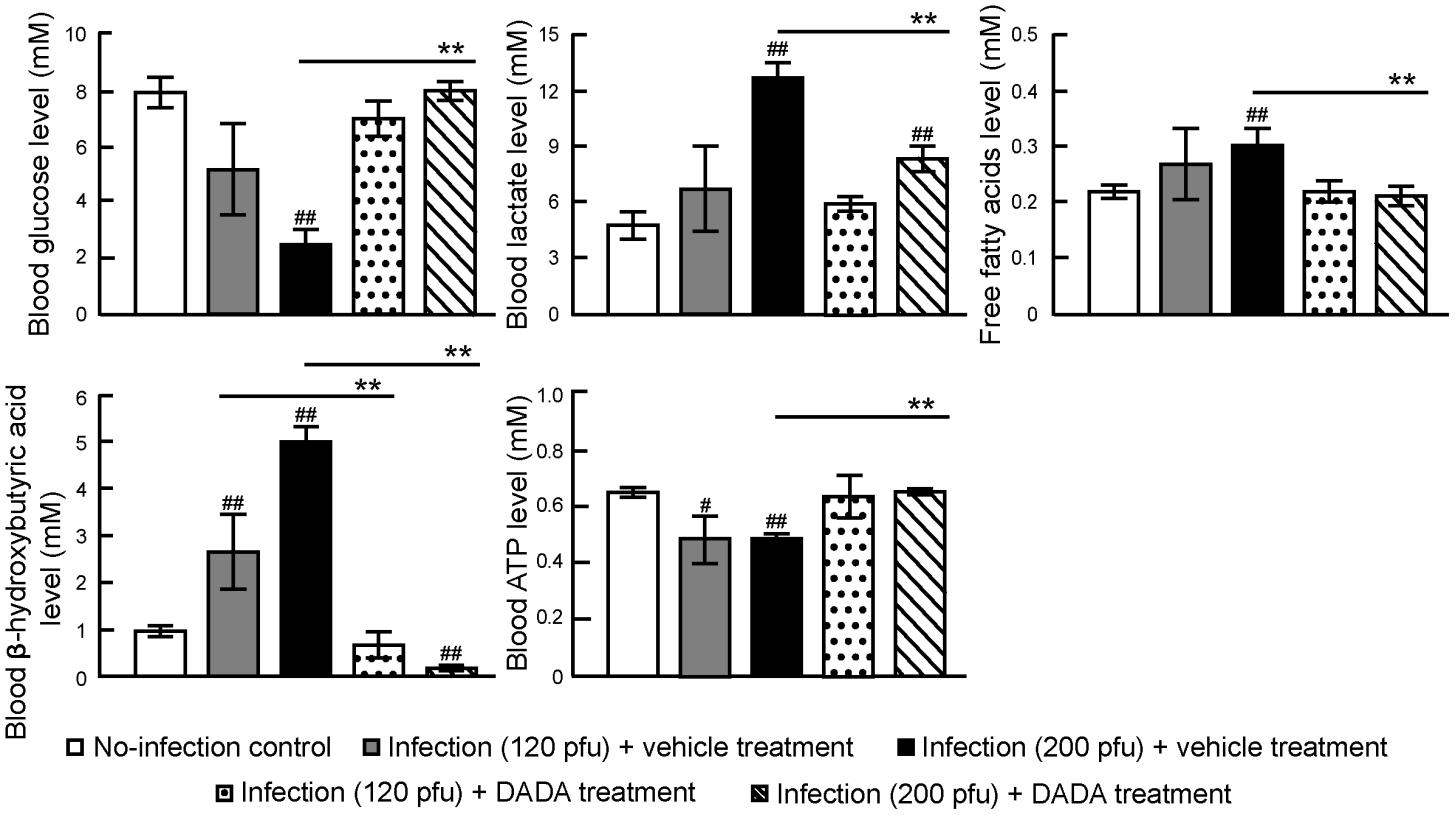
DADA improves blood glucose, lactate, β-hydroxybutyric acid, free fatty acids and ATP levels. Mice infected with 120 and 200 pfu of IAV were treated with oral DADA at 50 mg/kg or vehicle at 12-h intervals and the levels of glucose, lactate, free fatty acids, β-hydroxybutyric acid, and ATP in the blood were analyzed at day 7 post-infection. Values are mean ± SD of 5 mice per group. ^#^ *P*<0.05 and^##^ *P*<0.01 vs. no-infection. *P<0.05, **P<0.01, vs. infected group treated with vehicle, by one-way analysis of variance (ANOVA) and Tukey post hoc test.

Since PDH activity is closely linked to homeostasis of glucose, lipid, ketones, lactate and ATP [17]–[20], we next measured blood glucose, lactate, β-hydroxybutyric acid, free fatty acids and ATP levels at day 7 post-infection in untreated and DADA-treated mice infected with 120 pfu and 200 pfu of IAV (Figure 4). Mice infected with both doses of IAV showed decrease in glucose levels and increase in lactate, free fatty acids and β-hydroxybutyric acid levels in peripheral blood, with slight fall in blood ATP levels. These values tended to normalize after oral administration of DADA, and the effects of DADA were more remarkable in mice infected with 200 pfu of IAV than 120 pfu of IAV. Restoration by DADA in all blood metabolites analyzed in mice infected with 200 pfu of IAV was significant (*P*<0.01).

### DADA suppresses induction of proinflammatory cytokines

Experimental evidence suggests that metabolic disorders are tightly interconnected with inflammatory cytokines and adipokines [30]–[32]. Recent studies indicated that agonists of the peroxisome proliferation-activated receptor (PPAR)-γ, a lipid metabolite-mediated transcription factor, and agonists of AMP-activated protein kinase (AMPK), are safe and effective treatment regimens in patients with diabetes mellitus, significantly mitigate the IAV-induced immunopathology with cytokine storm as immunomodulatory agents, and protect mice against lethal IAV infection [33], [34]. These findings suggest that metabolic disorders induced by IAV infection are interconnected with cytokine up-regulation probably through metabolite-mediated signaling pathways and transcription factors.

**Figure 5:**
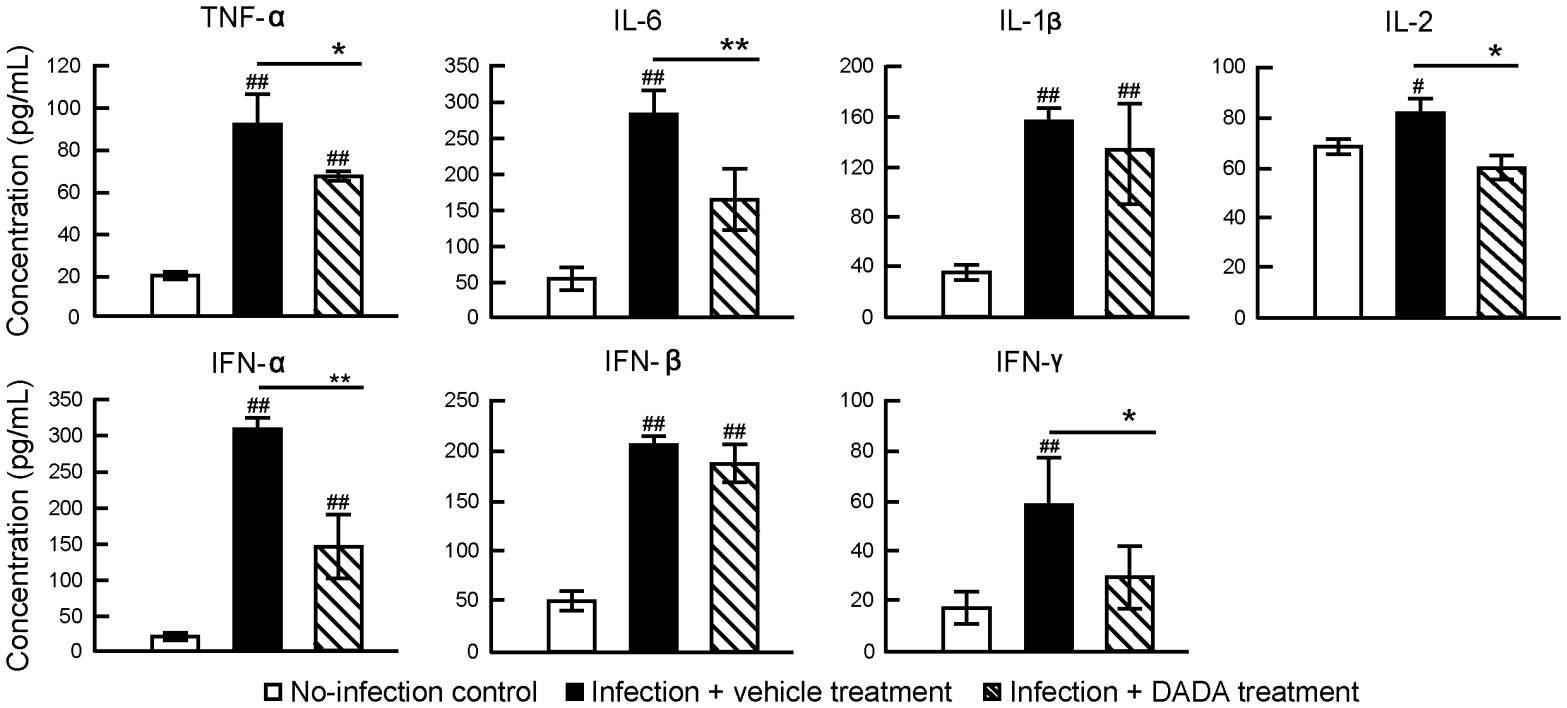
DADA suppresses induction of various cytokines in the lungs of IAV-infected mice. Mice infected with 120 pfu of IAV were treated with oral DADA at 50 mg/kg or vehicle at 12-h intervals and the levels of various cytokines in the lungs at day 2 post-infection were analyzed by ELISA. Data are mean ± SD of 5 mice per group. ^#^ *P*<0.05, ^##^ *P*<0.01, vs. no-infection, *P<0.05, **P<0.01, vs. infected group treated with vehicle, by one-way analysis of variance (ANOVA) with Tukey post hoc test.

Next, we assessed the effects of DADA treatment on cytokine levels at day 2 post-infection during cytokine storm duration in the lungs of mice [12], [35] infected with a sub-lethal dose of IAV. IAV infection significantly increased the levels of all tested proinflammatory cytokines (IL-1β, IL-6, IL-2, TNF-α, IFN-α, IFN-β and IFN-γ) in lung homogenates between 1.2- and 13.7-fold relative to the baseline (before infection) (Figure 5). Treatment with DADA significantly suppressed IL-6, IL-2, IFN-α, TNF-α and IFN-γ levels but not those of IFN-β and IL-1β.

### DADA reduces IAV replication in the lungs and trypsin expression in various organs

**Figure 6:**
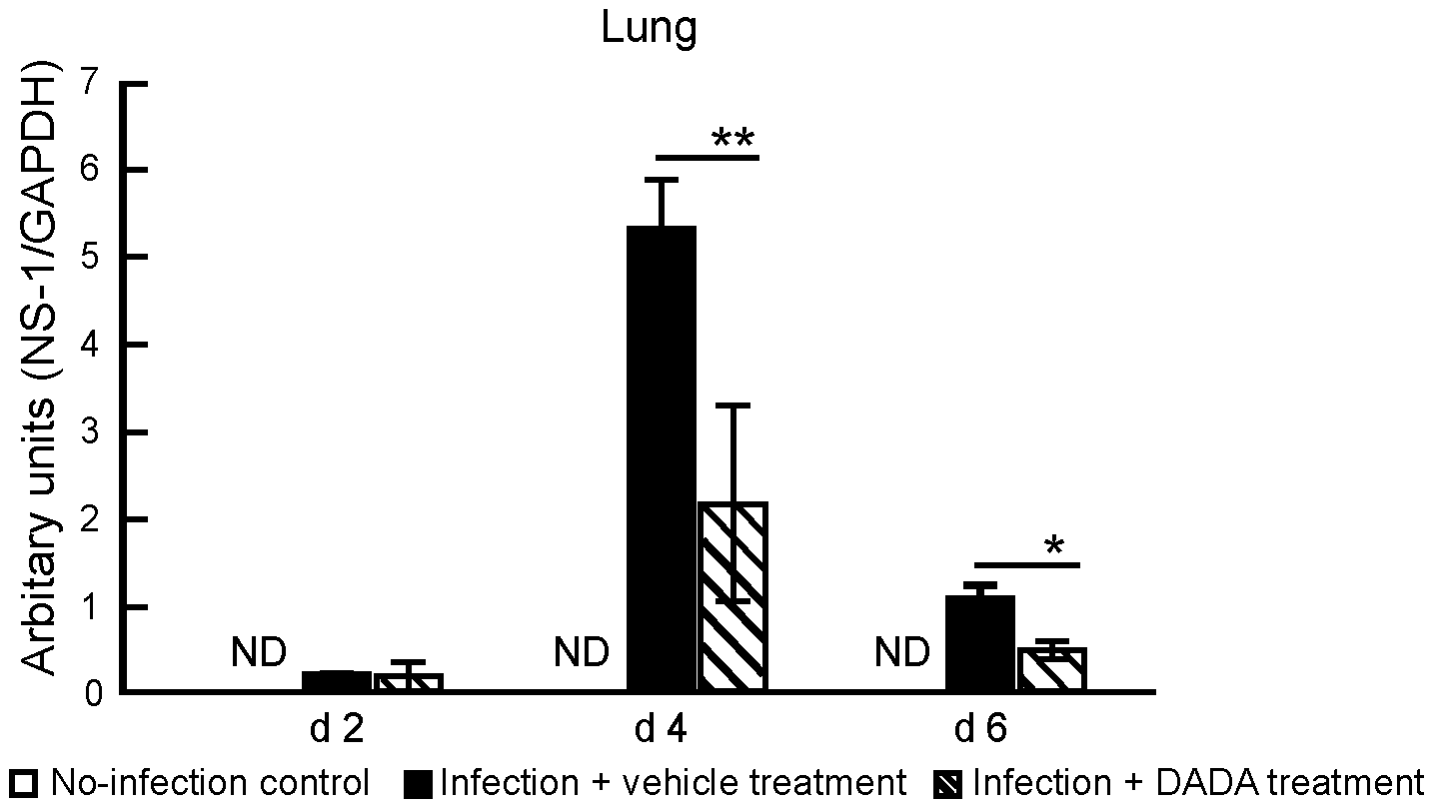
Effects of DADA on viral replication. Mice infected with 120 pfu of IAV were treated with oral DADA at 50 mg/kg or vehicle at 12-h intervals and viral NS1 replication in the lungs was analyzed quantitatively by real-time PCR at days 2 (d2), 4 (d4) and 6 (d6) post-infection. Data were normalized relative to GAPDH expression, which was used as the internal control. Open columns: control (no-infection) group [values are below the detection level (ND)]. Data are mean ± SD of three experiments in 3 mice per group. *P<0.05, **P<0.01, vs. infected group treated with vehicle, by one-way analysis of variance (ANOVA) and Tukey post hoc test.

**Figure 7:**
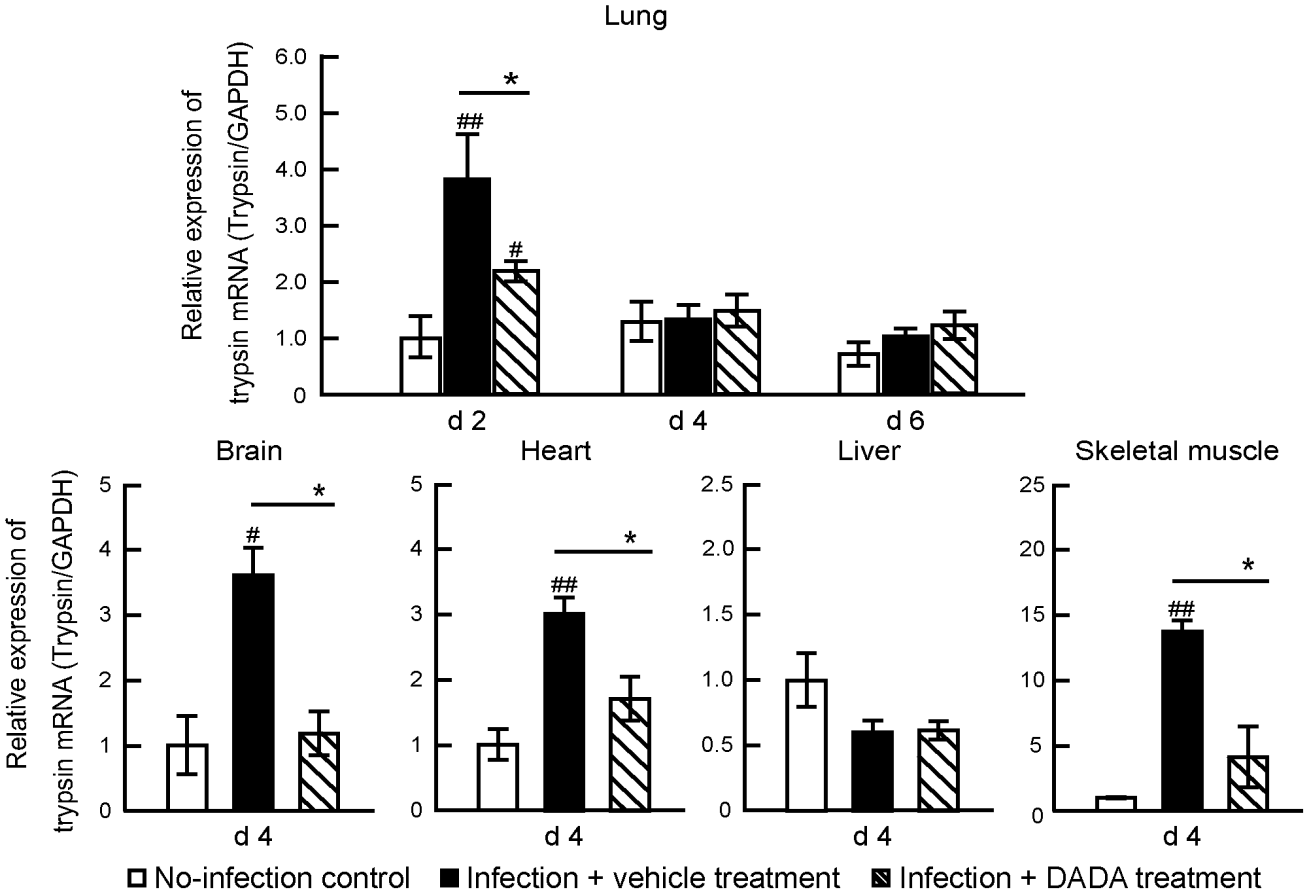
Effects of DADA on trypsin up-regulation. Mice infected with 120 pfu of IAV were treated with oral DADA at 50 mg/kg or vehicle at 12-h intervals and trypsin mRNA levels in the skeletal muscles, heart, liver and brain were measured by real-time PCR at day 4 post-infection. Trypsin mRNA levels in the lungs were also measured at days 2 (d2), 4 (d4) and 6 (d6) post-infection. Trypsin mRNA expression levels after IAV infection relative to that of no-infection. Values are mean ± SD of three experiments in 5 mice per each group. ^#^ *P*<0.05, ^##^ *P*<0.01, vs. no-infection, *P<0.05, **P<0.01, vs. infected mice treated with vehicle, by one-way analysis of variance (ANOVA) and Tukey post hoc test.

Since the levels of proinflammatory cytokines are closely interconnected to those of trypsin and influenza virus replication rate in target organs through the influenza virus–cytokine–trypsin cycle [7], [12], [13], we analyzed the effects of DADA on viral replication and trypsin expression in the lungs during the peak duration at days 2, 4 and 6 post-infection [7], [36], [37] and in other organs at day 4 post-infection [7], [13], [38]. Quantitative real-time PCR showed a significant reduction in the copy number of the viral non-structure protein 1 (NS-1) RNA gene segment in the lungs of infected mice treated with DADA at days 4 and 6 post-infection, compared with the vehicle-treated mice (Figure 6). Furthermore, the markedly induced trypsin levels in the lungs at day 2 post-infection and those in the skeletal muscles, heart and brain at day 4 post-infection were significantly suppressed in animals treated with DADA (Figure 7). However, IAV infection and DADA treatment had no significant effects on trypsin expression in the liver.

### DADA significantly increases survival rate of mice infected with semi- and sub-lethal doses of IAV and restores suppressed food and water intake after infection

**Figure 8:**
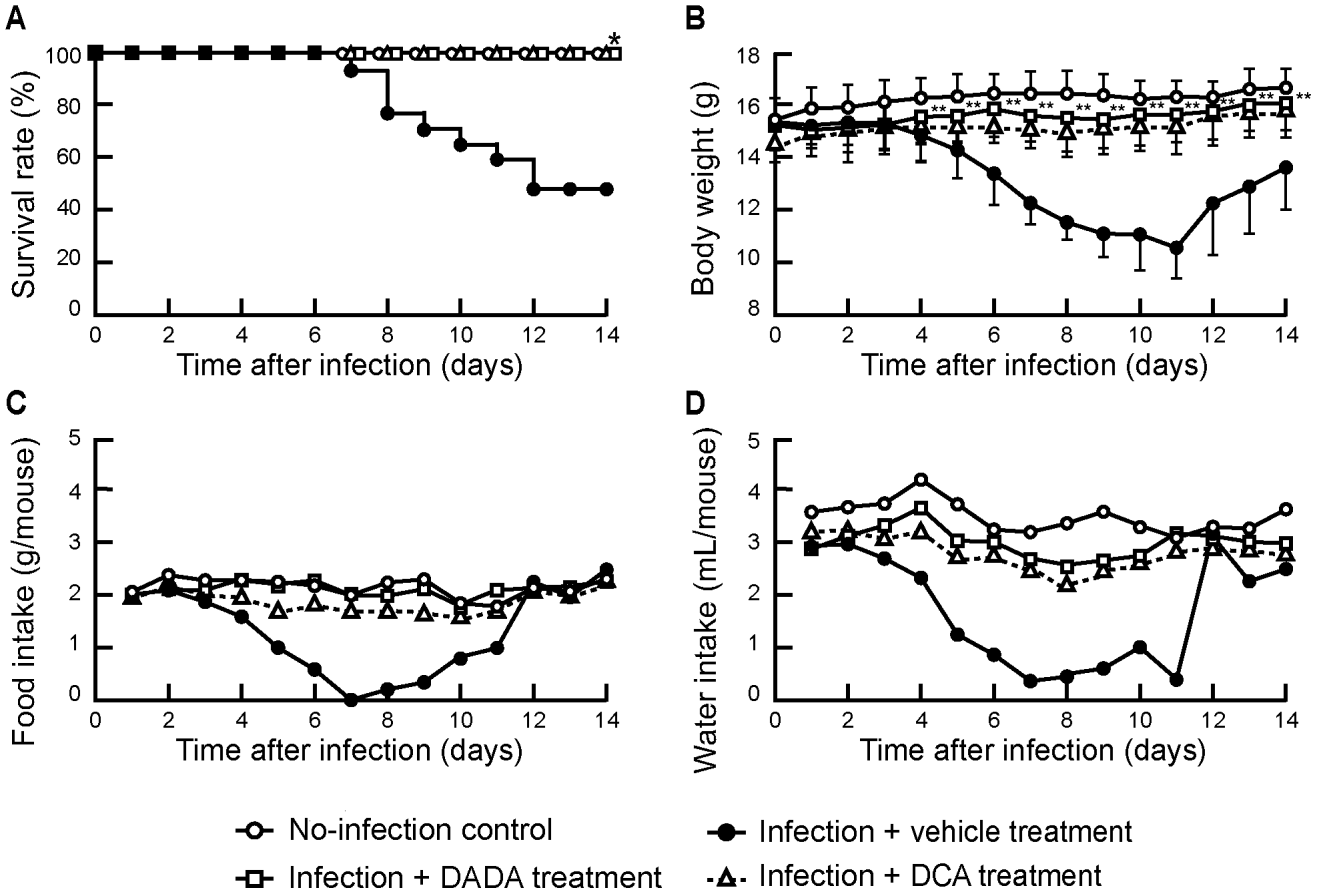
Effects of DADA on survival rate, body weight, and food and water intake. Mice infected with 60 pfu of IAV, representing the 50% lethal dose, were treated with oral DADA at 50 mg/kg, vehicle, or DCA administered peritoneally at 28 mg/kg at 12-h intervals for 14 days. The survival rate (A), body weight (B), food intake (C), and water intake (D) by infected mice were monitored. Differences in survival rate were analyzed by Kaplan-Meier and log-rank tests. Data are mean ± SD of 15 mice per group. *P<0.05, **P<0.01, vs. infected group treated with vehicle, by two-way ANOVA.

We also studied the effects of DADA on survival rate, body weight and food and water intake of mice infected with 60 and 120 pfu of IAV during the 14-day post-infection period. Mice infected with 60 pfu of IAV showed progressive avoidance of food and water during days 2 to 7 post-infection, and the animals died after infection for 7 days (Figure 8). The body weight started to fall one day after the reduction in food and water intake. However, DADA-treated infected mice showed no significant decrease in food and water intake as well as no significant reduction in body weight during the 14-day experimental period. While infected mice showed continuous decrease in the survival rate after day 7, with a survival rate of 50% at day 14 post-infection, none of the DADA-treated mice died during the experimental period. The effects of DCA administered at an equivalent molar dose to DADA, on survival rate and body weight of infected mice paralleled those of DADA during the experimental period. The time courses of avoidance of food and water of mice infected with 120 pfu of IAV were similar to those in mice infected with 60 pfu of IAV. Although mice showed continuous decrease in the survival rate after day 7 post-infection to 5% at day 14 post-infection, DADA treatment significantly reduced mortality from 95% to 50% (*P*<0.05) (data not shown).

### DADA improves pathological changes in the lungs after infection

**Figure 9:**
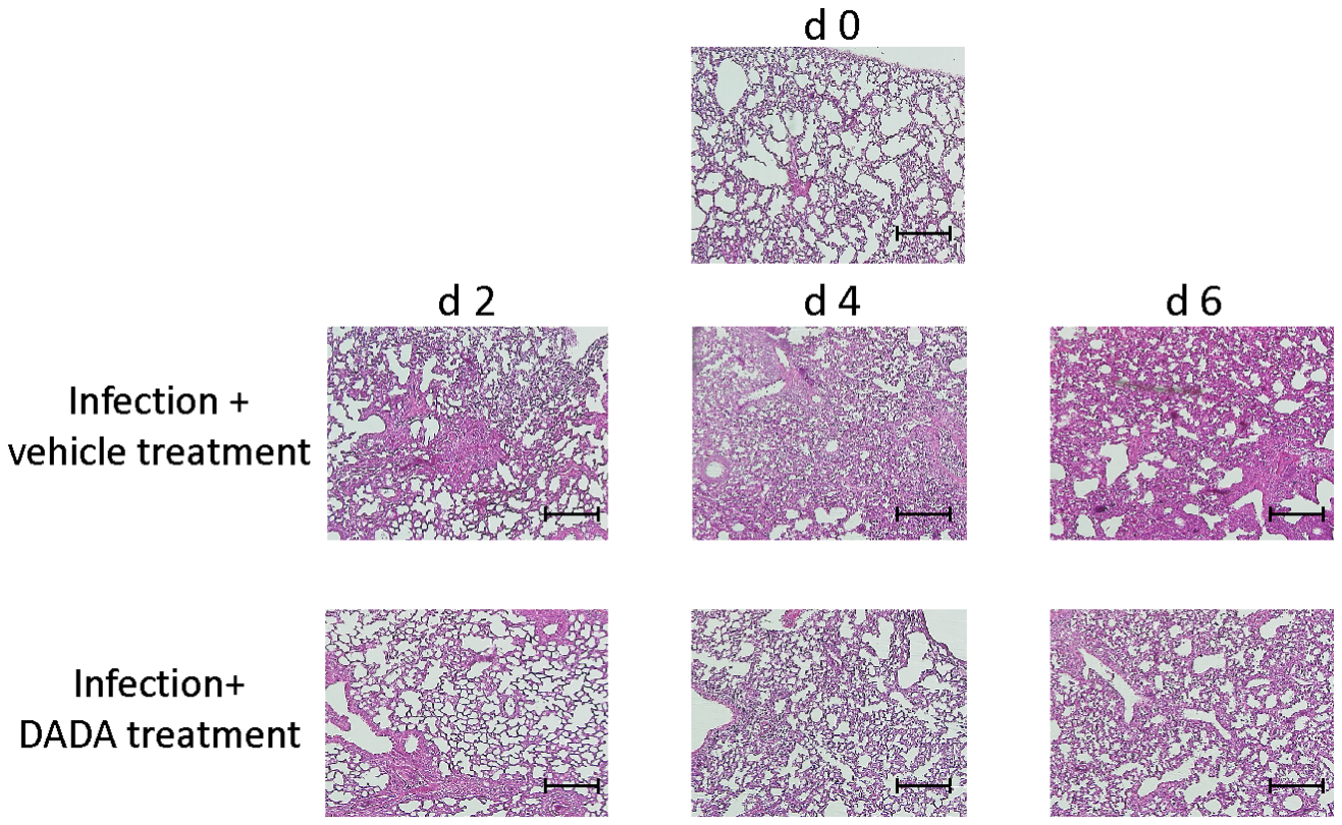
Effects of DADA on pathological changes in the lungs. Mice infected with 60 pfu of IAV were treated orally with DADA at 50 mg/kg or vehicle at 12-h intervals for 6 days. The lungs were isolated at days 0 (d0), 2 (d2), 4 (d4) or 6 (d6) post-infection, processed and stained with hematoxylin and eosin for histopathological evaluation. Each result is representative of 5 animals in each group. Bar, 300 µm.

Finally, we prepared histological sections of the lungs of mice infected with 60 pfu of IAV to confirm the effects of DADA at days 0, 2, 4 and 6 post-infection using hematoxylin and eosin-stained sections of the lungs (Figure 9). In infected animals treated with vehicle, focal inflammatory infiltrates were noted in the lungs at day 2 post-infection, followed by progressive and extensive infiltration throughout the entire lungs at days 4 and 6 post-infection. DADA treatment effectively suppressed the inflammatory infiltration during days 0 and 6 post-infection, although mild infiltrates were detected at days 4 and 6 post-infection (Figure 9).

## Discussion

The present study reported several new findings: (i) Severe IAV infection markedly up-regulated PDK4, together with down-regulation of energy homeostasis and disorders of glucose and lipid metabolism. The results suggest that PDK4 is a sensitive biomarker of MOF and a target molecule for treatment in severe IAV infection. (ii) DADA effectively inhibited PDK4, with inhibition kinetics similar to DCA. (iii) DADA-induced PDK4 inhibition effectively suppressed IAV-induced MOF *in vivo*, with amelioration of suppressed energy homeostasis, disorders of glucose and lipid metabolism and cytokine up-regulation, and suppressed viral replication. The results suggest that PDK4 is a potentially suitable therapeutic target for MOF in severe IAV infection.

Until recently, most influenza virologists have concentrated on the development of therapeutic strategies that target virus replication [39]. However, MOF starts just after the peak of viral proliferation with the induction of host cellular defense system, such as innate and adaptive immune responses [12]. Furthermore, anti-viral neuraminidase inhibitors do not offer effective treatment for MOF after viral proliferation. Previous studies on the potential therapies proposed the use of various immunomodulatory agents, such as agonists of PPARγ and AMPK, with the potential of mitigating the effects of IAV-induced cytokine storm [33], although the precise mechanisms of the therapeutic actions of these agents remain so far elusive. On the other hand, studies on the pathogenesis of MOF discussed the influenza virus–cytokine–trypsin cycle as the pathomechanism of vascular hyperpermeability in MOF with cytokine storm in severe IAV infection and influenza-associated encephalopathy [5], [7], [13]. Upregulation of TNF-α alters the cellular redox state through its receptor and reduces ATP synthesis in mitochondria [8], [9], resulting in increased cellular junctional permeability [11]. In addition, IAV infection and/or the related proinflammatory cytokine induction results in upregulation of trypsin, which induces marked increase in [Ca^2+^]_i_ and loss of zonula occudens-1 in endothelial cells via the protease-activated receptor 2 signaling [7]. Since fatty acid oxidation is a major energy pathway for ATP generation in mitochondria of endothelial cells [40], [41], cells with disordered lipid metabolism suffer ATP depletion and dissociation of junctional complexes with increased permeability [42], [43]. These results suggest that the lesser fuel utilization by the thermolabile compound variants of CPT II, a pivotal component of ATP generation through mitochondrial long-chain fatty acid oxidation, is an important etiological cause of acute brain edema and MOF during high fever in patients with influenza-associated encephalopathy [5], [15], [44].

**Figure 10:**
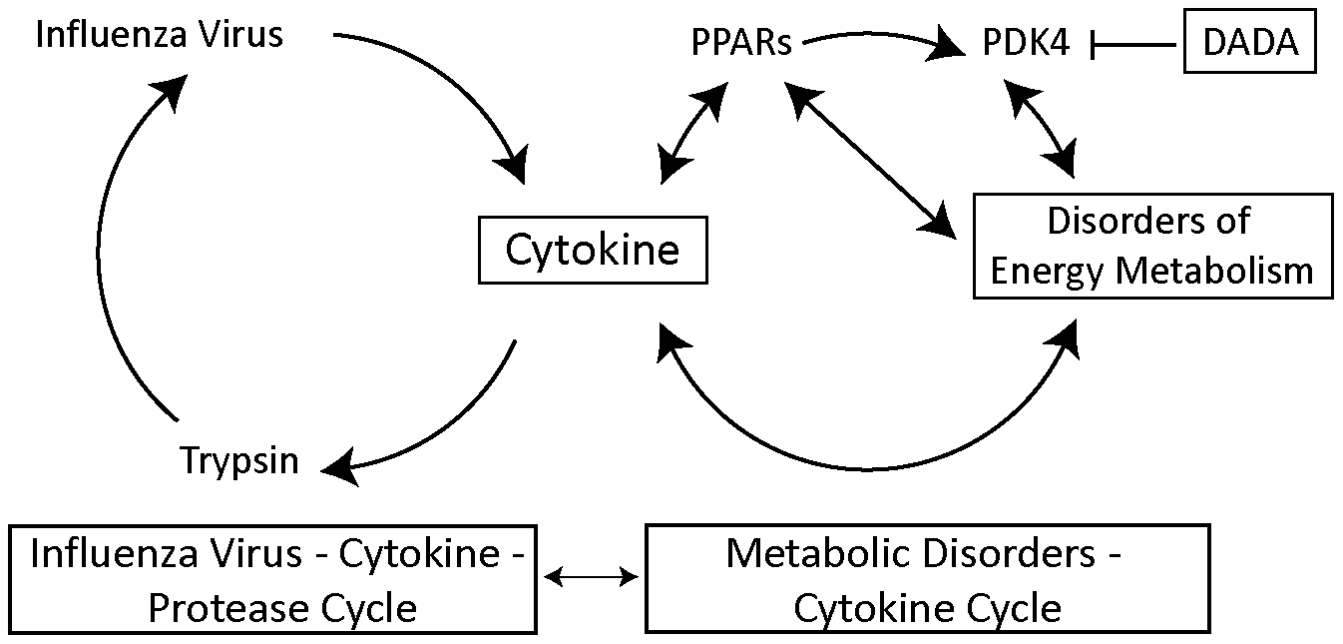
Diagram illustrating the proposed pathogenic mechanism of MOF in severe influenza involving the host metabolic disorders–cytokine cycle linked to the influenza virus–cytokine–trypsin cycle. PPARs, peroxisome proliferator-activated receptors; PDK4, pyruvate dehydrogenase kinase 4; DADA, diisopropylamine dichloroacetate.

As an extension to our previous studies, we added in the present study a new evidence for the marked up-regulation of PDK4 in IAV infection with suppression of PDH activity and ATP levels in the mitochondria, in addition to metabolic disorders and MOF in mice. It is well known that PDK4 is up-regulated by low food intake [18] and the pathological findings induced by IAV infection may be the complex outcome of viral proliferation, cytokine storm and PDK4 up-regulation associated with the lack of food intake after infection. The expression of PDK4 is transcriptionally regulated by PPARs (PPARα, PPARβ/δ, and PPARγ) in a tissue-specific manner [17], [20], [45], [46]. Moseley and colleagues [33] reported that PPARγ and AMPK agonists provide protection in mice infected with highly pathogenic and pandemic strains of IAV. In the skeletal muscles and liver, the major resting energy expenditure organs in mammals [47], treatment with PPARγ and AMPK agonists reduces PDK4 expression in these organs, which increases glucose utilization and decreases the expression of genes involved in fatty acids transport and oxidation [45], [48], [49], although PPARγ agonists result in opposite increase in PDK4 expression in white adipose tissue [20]. These results support our finding that PDK4 inhibition by DADA and DCA significantly improved the survival of mice infected with severe IAV (Figure 8). Based on our new findings, together with the growing evidence of interrelation between cytokines and metabolic disorders [30]–[32], interrelation between cytokines and PPARs [33], [46], [50], and interrelation between PPARs and metabolic disorders through PDK4 [50]–[52], we propose a new concept of host cellular mechanism for MOF in severe influenza (Figure 10). This concept involves the metabolic disorders–cytokine cycle, which is interconnected with metabolite-mediated signaling pathways and transcription factors, such as PPARs, and PDK4, and in turn is closely linked to the influenza virus–cytokine–trypsin cycle for viral proliferation through cytokines. Coupling of these two cycles can explain the actions of DADA, through PDK4 inhibition, on normalization of glucose and lipid metabolism and ATP levels in the mitochondria, as well as suppression of cytokine production and viral replication in the lungs and trypsin up-regulation in various organs of mice infected with severe IAV (Figures 3–7), with resultant improvement in the survival rate (Figure 8).

Recent studies have demonstrated the involvement of mitochondria in a broad range of innate immune pathways, apart from the control of apoptosis, during infection [53]. Signaling molecules of antiviral immunity, such as mitochondrial antiviral signaling protein, IFN-β promoter stimulator 1, caspase-recruitment domain adaptor inducing IFN-β, and virus-induced signaling adaptor, are present on mitochondrial membranes and their activities are regulated by membrane potential (ΔΨ_m_), ATP level and reactive oxygen species in the mitochondria [54]. Although the interaction between PDK4 and these signaling molecules on the membranes of mitochondria has not been elucidated so far, restoration of suppressed mitochondrial ATP levels and ΔΨ_m_ in severe IAV infection by PDK4 inhibitors may augment mitochondria-derived antiviral immunity, resulting in suppression of IAV replication (Figure 6).

DADA-induced PDK4 inhibition produced marked suppression of IFN-α induction, but it caused only mild and insignificant inhibition of IFN-β (Figure 5). How can one explain these effects even though both cytokines are produced by the same type of cells and induced markedly and equally by IAV infection? While we cannot provide a solid explanation for this finding, the difference in DADA suppressive capacity on IFN-α and IFN-β may be due to the indirect inhibitory effects of DADA on cytokine production (see Figure 10). Further studies are required to determine the precise mechanisms of action of DADA on cytokine suppression.

Only the brain, among all the organs investigated in the present study, showed very mild or no pathological changes in PDH, PDK4 and ATP, except trypsin, in severe IAV infection. This sparing effect is probably related to the protective role of the blood-brain barrier against the transport of pathogenic molecules into the brain. However, trypsin in the brain is distributed mainly in cerebrovascular endothelial cells and hippocampal neuronal cells [12], [38], and it is up-regulated in vascular endothelial cells by proinflammatory cytokines in the blood [7]. These findings suggest that the high levels of proinflammatory cytokines in the blood after IAV infection induce trypsin in cerebrovascular endothelial cells and DADA may suppress trypsin levels in the brain through down-regulation of cytokines. On the other hand, IAV infection and DADA treatment had no significant effects on trypsin expression in the liver (Figure 7). The findings may be related to the high expression of anti-trypsin in the liver [55] and its effect of trypsin inhibition on the influenza virus–cytokine–trypsin cycle.

Among the known synthetic inhibitors of PDK, the pyruvate analog DCA is the most common classic inhibitor of PDK isoforms, including PDK4 [29], [56]. DCA is known to have beneficial effects in inborn errors of mitochondrial metabolism, diabetes, lactic acidosis, myocardial ischemia and metabolism of cancer cells to increase mitochondrial-dependent apoptosis [29], [57]–[59]. However, DCA also has symptomatic adverse reaction of peripheral neuropathy [23], [60], [61]. In the present study, DADA, a new PDK4 inhibitor, inhibited PDK4 and PDK2 with potency similar to DCA (Table 3). DADA was originally obtained from apricot nuclei and called pangamic acid or “vitamin B15” [21] and has been used as a safety treatment option for hepatic injury and nonalcoholic fatty liver diseases [62] without symptomatic peripheral neuropathy or other adverse effects. The present study suggests that DADA is an alternative and safe PDK4 inhibitor, potentially suitable for treatment of MOF in severe IAV infection.
